# The Clinicopathological Significance of MicroRNA-155 in Breast Cancer: A Meta-Analysis

**DOI:** 10.1155/2014/724209

**Published:** 2014-08-03

**Authors:** Hui Zeng, Cheng Fang, Seungyoon Nam, Qing Cai, Xinghua Long

**Affiliations:** ^1^Zhongnan Hospital of Wuhan University, Wuhan 430071, China; ^2^Cancer Genomics Branch, National Cancer Center, Goyang-si, Gyeonggi-do 410-769, Republic of Korea; ^3^Hubei University of Chinese Medicine, Wuhan 430061, China

## Abstract

*Objective*. Previous studies demonstrated that the associations between expression level of microRNA-155 (miR-155) and clinicopathological significance of breast cancer remained inconsistent. Therefore, we performed a meta-analysis based on eligible studies to summarize the possible associations. * Methods*. We identified eligible studies published up to May 2014 by a comprehensive search of PubMed, EMBASE, CNKI, and VIP databases. The analysis was performed with RevMan. 5.0 software. * Results.* A total of 15 studies were included. The results of meta-analysis showed that miR-155 was positively correlated with breast cancer with standardized mean difference (SMD) = 1.22. Elevated miR-155 was found in Her-2 positive or lymph node metastasis positive, or p53 mutant type breast cancer. But the result showed to be insignificant in TNM comparison. With respect to estrogen receptor alpha (ER) and progesterone receptor (PR) status, both of them showed significant associations with SMD = −1.2 and −1.85, respectively.* Conclusion. *MiR-155 detection might have a diagnostic value in breast cancer patients. It might be used as an auxiliary biomarker for different clinicopathological breast cancer.

## 1. Introduction

Development of tumors is considered to be a complex multistep process, including the activation of oncogenes and the silencing of tumor suppressors [1]. MicroRNAs (miRNAs) are kinds of highly conserved, approximately 20~22 nucleotide noncoding RNAs. Endogenous miRNAs inhibit the expression of target genes in the translation level by assembling to RNA-induced silence complex (RISC) through parts of base pairing with the 3′ untranslated region (UTR) of target mRNAs [2–4].

Recent studies have shown that miRNAs were involved in various physiological and pathological processes, and an increasing number of evidences prove that the abnormal expression of miRNAs has a direct correlation with the occurrence and development of cancers. The abnormal expression and dysfunction of miRNAs may lead to cellular disorder and finally result in diseases or even cancers [5]. Thus miRNAs may be exploited as new kinds of molecular targets for diagnosing and treating cancers.

At present, in studies that focused on the association between miRNAs and tumors, abnormal expression of miR-155 in patients with breast cancer has gained substantial attention. As an oncogene, high expression of miR-155 was considered as a breast cancer risk factor. Suppressor cytokine signaling-1 (*socs1*) is a constant target gene of miR-155 in breast cancer cells in the human evolutionary process. The expression of miR-155 and socs1 is negative correlated [6]. The study by Kong et al. has shown that miR-155 participates in the process of epithelial to mesenchymal transition (EMT) and infiltration in NMuMG cells [7], suggesting that miR-155 could be used as a diagnostic marker for breast cancer metastasis. Our group has reported multiple miRNA network clusters involving in antiestrogen resistance in breast cancer; miR-155 was one of the most important dysregulated miRNAs, indicating that miR-155 might be utilized as a diagnostic marker for breast cancer endocrine therapy [8].

As the most common malignant tumor in the world among women, it had been anticipated that there would be 1.5 million women being newly diagnosed by 2010 in 2004 [9]. In Chinese population, breast cancer has an incidence rate of 16.39 per 100000 Chinese women and seriously affects people's lives and health. Among cancers that happened in Chinese women of economically developed provinces and cities, breast cancer has the highest incidence and is the fourth most common cause of cancer death [10]. Recently, there were many investigations referring to the relationship between miR-155 and breast cancer, but because of the small sample size, discrepancy exists among different studies more or less. Therefore, in order to provide a more considerable, clearer, and more systematic recognition of the expression of miR-155 in breast cancer, we collected the data related to miR-155 expression in breast cancer to carry out the meta-analysis.

## 2. Materials and Methods

### 2.1. Search Strategy

The literature retrieval was performed by two independent reviewers (Hui Zeng and Cheng Fang). All studies included in the meta-analysis were selected by searching the PubMed, EMBASE, CNKI and VIP databases up to May 2014 using the following keywords: “miR-155” or “microRNA-155” and “breast cancer.” All references in these studies were examined to identify additional research that was not indexed by the databases. We selected published articles written in English or Chinese.

### 2.2. Including and Excluding Criteria

Including criteria include (1) being related to breast cancer and miR-155, (2) patients that are confirmed by pathology, (3) sufficient data, including mean value and standard deviation or other data that can result in mean value and standard deviation, and (4) measurement methods and experiment group that are the same or almost same.

Excluding criteria include (1) reviews, comments, or letters, (2) low-quality or incomplete data, (3) abstract only and lack of the full text, without author's reply, and (4) reduplicate publication. For duplicate articles, only the most recent or largest data set was selected.

### 2.3. Data Extraction

Data were extracted from eligible articles independently by two of the authors, with any disagreement resolved by consensus. The following information was collected in a predefined data collection form: first author's name, publication year, country, sample type, total number of cases and controls, quantitative methods, and publication language.

### 2.4. Literature Quality Evaluation

Based on the results of the system, we used grading method [11] recommended by the GRADE system to evaluate quality of evidence. Evidence quality classification is as follows: (I) high quality: further research would not change the credibility of evaluation results about the curative effect; (II) medium quality: further research is likely to affect the credibility of evaluation results about the curative effect and may change the assessment results; (III) low quality: further research is likely to affect the credibility of evaluation results about the curative effect, and the assessment results are very likely to change; (IV) very low quality: any curative effect evaluation results are uncertain.

### 2.5. Statistic Analysis

Rev-Man 5.0 software which was recommended by Cochrane collaboration was used in this meta-analysis. Rev-Man which is short for review manager is the software used for preparing and maintaining Cochrane reviews. Results can be presented graphically with the software.

Firstly, heterogeneity between studies was assessed by *χ* ^2^-based *Q*-tests and *I* ^2^ tests, where *I* ^2^ (%) > 50% or *P* < 0.10 was considered significantly heterogeneous [12]. The random effect model (DerSimonian-Laird) [13] was used to assess pooled odds ratios (ORs) when significant heterogeneity was observed. Otherwise, the fixed effect model (Mantel-Haenszel) [14] was used. Sensitivity analysis was used to analyze the stability of the text results by omitting one study at a time. For continuous data, if quantitative method is the same, we adopted the weighted mean difference (MD) as our analysis index. If they used different measuring instruments or units for the same variable or there was large difference among the mean value of numerical analysis, the standardized mean difference (SMD) was adopted for analysis. We calculated 95% confidence interval (CI) of all analysis. At the same time, the funnel chart is used to determine publication bias. Data input and monitor were done by two researchers.

## 3. Results

### 3.1. Study Characteristics

**Figure 1:**
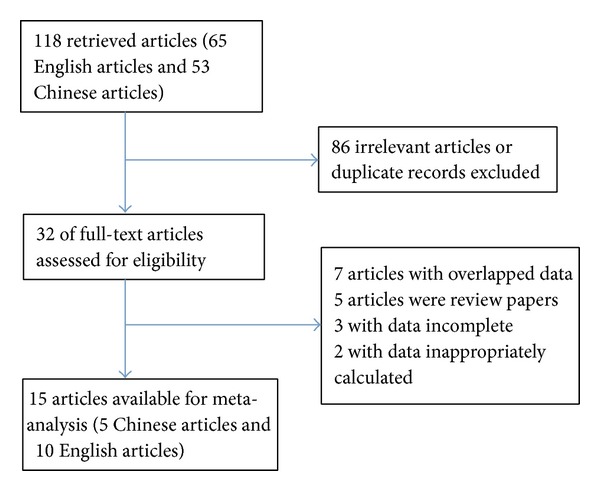
Flow diagram of study identification.

For the initial inspection, 65 related English articles and 53 Chinese articles were obtained by literature search from the PubMed, EMBASE, CNKI, and VIP databases. After titles and abstracts were screened, 86 articles were excluded because of irrelevant or duplicate records. The full texts of the remaining 32 records were carefully reviewed. Among these articles, seven articles were abandoned for overlapped data, and five articles were excluded because of review papers. Another five were excluded due to data that were incomplete or inappropriately calculated or original data that could not be obtained despite attempts to contact the authors. Therefore, 15 [15–29] articles were considered in the present meta-analysis. Among them, 13 discussed the different expression level of miR-155 between breast cancer samples and normal samples, and another 2 only discussed it between different subtype breast cancers. One [27] was included in ER and PR analysis, and another [28] was included in TNM and p53 analysis. The specific retrieval process was shown in Figure 1. The study characteristics included in the meta-analysis were listed in Table 1.

### 3.2. Results of Meta-Analysis

**Figure 2:**
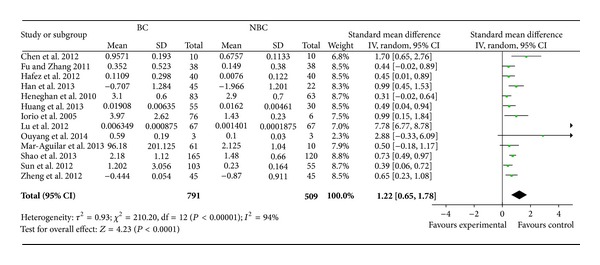
Random effects standardized mean difference (SMD) for the association of miR-155 expression level and breast cancer. BC: breast cancer and NBC: nonbreast cancer. The central of the square means the study-specific SMD and the horizontal lines correspond to the study-specific 95% CI.

A total of 13 studies including 791 breast cancer samples and 509 normal samples were collected in this section. As shown in Figure 2, we observed an elevated miR-155 expression in breast cancer samples (SMD = 1.22, 95% CI = 0.65–1.78, *P* < 0.00001).

**Figure 3:**
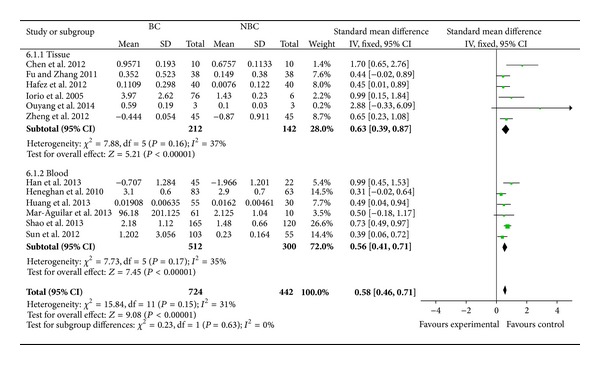
Fixed effects standardized mean difference (SMD) for the association of miR-155 expression level and subgroup breast cancer. BC: breast cancer and NBC: nonbreast cancer. The central of the square means the study-specific SMD and the horizontal lines correspond to the study-specific 95% CI.

However, high heterogeneity was observed in the analysis, and sensitivity analyses indicated that the study from Lu et al. [22] was mainly responsible for the observed heterogeneity. When we excluded this study, the high heterogeneity was significantly decreased and the association was still significantly different (for SMD = 0.58, 95% CI = 0.46–0.71, *P* < 0.00001, *P* for heterogeneity = 0.15, *I* ^2^ = 31%). Sensitivity analyses indicated that the pooled SMD was consistently significant by omitting one study at a time for the last studies. We then did a subgroup analyses to investigate the expression level of miR-155 in different sample types. As the study of Lu et al. [22] mainly contributed to the high heterogeneity, we excluded it in the subgroup analyses. In both tissue sample and blood sample, higher miR-155 expression level was detected in breast cancer samples than that in normal samples (with SMD = 0.63, 95% CI = 0.39–0.87, *P* < 0.00001, SMD = 0.56, 95% CI = 0.41–0.71, *P* < 0.00001, resp., Figure 3).

In subtype analysis, the expression level of miR-155 was significantly correlated with estrogen receptor alpha (ER), progesterone receptor (PR), Her-2, lymph node metastasis, tumor size, and p53 status. In subtype analysis of different ER and PR status breast cancer, miR-155 was significantly less expressed in ER+ or PR+ breast cancer. But it was highly expressed in Her-2+ or lymph node metastasis positive breast cancer compared with Her-2− or lymph node metastasis negative breast cancer. Comparing breast cancer with tumor size >2 cm with that <2 cm, the one with larger tumor may be along with higher miR-155 expression level. When breast cancer with wild p53 type and breast cancer with mutant p53 type were compared, higher miR-155 expression was detected in mutant p53 type breast cancer. However, the miR-155 expression level in different TNM grades breast cancer samples showed no significant difference (Table 2).

### 3.3. Evaluation of Publication Bias

**Figure 4:**
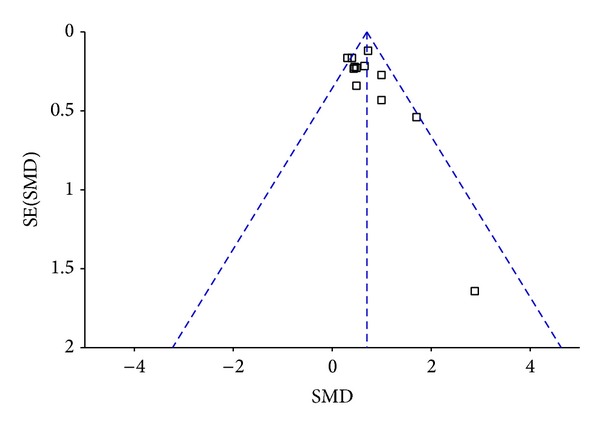
Funnel plots of studies included in the overall analysis.

We assessed the publication bias of the literature by the funnel plot. The funnel plot showed that most of the researches lay in the top of the funnel and rare in the base. The shape of the funnel plot did not reveal any evidence of obvious asymmetry (Figure 4). Egger's test and Begg's test showed *P* > 0.05.

## 4. Discussion

Although the pathogenesis of breast cancer has not been completely understood, the occurrence, development, and metastasis of cancer are sure to be genes participated. In recent years, miRNA studies started a new field for cancer research; there were studies showing [21, 30–32] that miR-155 might be closely related with breast cancer and played a crucial role in the development of it. Zhu et al. [33] found that miR-155 expression in breast cancer tissues was higher than that in normal tissue, lymph node metastasis and the level of estrogen receptor alpha (ER) and progesterone receptor (PR) were associated with the expression of miR-155 level in the study. Wang et al. [27] also showed significant associations between the expression of miR-155 level and the status of ER and PR. In this meta-analysis, we analyzed the correlation between expression level of miR-155 and characteristics of breast cancer. We did our best to do a comprehensive search to avoid publication bias and closely followed both including and excluding criteria. We evaluated the publication bias by using the Egger's test and Begger's test, resulting in *P* > 0.05. It indicated that no significant publication bias existed. In the analysis of the homogeneity of the literatures that had been incorporated, we found that most had heterogeneity. In this case, we preferred random effects model.

According to the results, we found that the expression level of miR-155 in breast cancer sample was greater than that in nonbreast cancer sample. Results of most articles reported are almost consistent. Liu et al. [34] showed in their article that the expression level in breast cancer group was 3.2-fold higher than that in nonbreast cancer group. Other studies also have shown the miR-155 expression in breast cancer sample are at least 2-fold higher than that in nonbreast cancer sample; for example, Hui et al. [35] reported 2.4-fold overexpression in breast cancer; Sun et al. [26] reported 2.94-fold overexpression. When analyzing the different expression folds of miR-155 in breast cancer samples relative to normal samples of available data [6, 17, 26, 34–37], we discovered that the expression folds of miR-155 in breast cancer samples were higher than that in normal samples with the average expression about 6 times folds. In the subgroup analysis by sample type, the associations between miR-155 expression level and breast cancer showed to be significant in both tissue and blood sample. Therefore the detection of the expression level of miR-155 in blood can be used in diagnosing breast cancer as an auxiliary molecular marker.

According to our analysis, the higher expression level of miR-155 in breast cancer samples than that in normal samples was detected. However, the level in different clinical pathology breast cancer samples also showed to be inconsistent. So we merged into available literatures for the analysis. Pooled results for different subtypes showed that the expression level of miR-155 was not statistically significant associated with TNM-staging, but the expression level of miR-155 in lymph node metastasis positive group was significantly higher than that in lymph node metastasis negative group.

ER and PR were proteins which played important roles in the regulation of the growth and differentiation of breast cancer [38]. According to our analysis, significantly higher expression level of miR-155 was detected in both ER- and PR- group.

Gene p53, as a tumor suppressor, is located on the short arm of chromosome 17. It can inhibit cell transformation and activity of cancer gene. But mutant gene p53 can cause cell transformation, unlimited cell growth, and cancer. Gene p53 mutation may be the most important deterioration factor of breast cancer [39]. In the study, it showed that miR-155 was significantly overexpressed in breast cancer with p53 mutant type compared with breast cancer with p53 wild type.

Her-2 is a prooncogene which is also located on chromosome 17. It is recognized to be one of the most closely related gene to breast cancer. MiR-155 is overexpressed in Her-2+ breast cancer compared with Her-2− breast cancer.

Although our meta-analysis represented a quantified synthesis of all available studies, some limitations should be noticed. First, this meta-analysis was conducted based on case-control studies, which might encounter recall and selection bias. Second, in subgroup analyses by sample type and subtype analyses, the number of the studies was relatively small. A further analysis in the subtype analysis could not be performed. Third, lack of the original data of available studies limited our further evaluation of potential associations.

In conclusion, we can demonstrate that miR-155 is one of the most significant altered miRNAs in breast cancer. And the overexpression of miR-155 is not so related to TNM stage, but it is closely related to lymph node metastasis, p53 status, and hormone receptor status of breast cancer patients. More sizable sample based clinical investigations should be conducted before miR-155 can be applied as an auxiliary diagnostic biomarker.

## Figures and Tables

**Table 1:** Characteristics of studies included in this meta-analysis.

Reference	Year	Origin	Sample size (case/control)	Quantitative method	Language
Chen et al. [15]	2012	China	Tissue 92/92	qRT-PCR	English
Fu and Zhang [16]	2011	China	Tissue 38/38	qRT-PCR	Chinese
Hafez et al. [17]	2012	Egypt	Tissue 40/40	qRT-PCR	English
Han et al. [18]	2013	China	Serum 45/22	qRT-PCR	Chinese
Heneghan et al. [19]	2010	Ireland	Blood 83/63	qRT-PCR	English
Huang et al. [20]	2013	China	Plasma 55/30	qRT-PCR	Chinese
Iorio et al. [21]	2005	Italy	Tissue 76/6	microarray	English
Lu et al. [22]	2012	China	Tissue 67/67	qRT-PCR	English
Ouyang et al. [23]	2014	China	Tissue 3/3	microarray	English
Mar-Aguilar et al. [24]	2013	Mexico	Serum 61/10	qRT-PCR	English
Shao et al. [25]	2013	China	Serum 165/120	qRT-PCR	Chinese
Sun et al. [26]	2012	China	Serum 103/55	qRT-PCR	English
Wang et al. [27]	2010	China	Tissue 58/58	qRT-PCR	English
Wang and Zhang [28]	2011	China	Serum 20/10	qRT-PCR	Chinese
Zheng et al. [29]	2012	China	Tissue 45/45	qRT-PCR	Chinese

**Table 2:** Analysis of miR-155 expression level and different subtype breast cancer.

Comparisons	*N*	Heterogeneity		Model^a^	Effect size	
	Studies	*I* ^2^ (%)	*P*(*Q*)		SMD	*P*(*Z*)
ER+ Versus ER−	7 [16–18, 20, 22, 27, 29]	96	<0.00001	R	−1.2	0.03
PR+ Versus PR−	7 [16–18, 20, 22, 27, 29]	97	<0.00001	R	−1.85	0.01
Her2+ Versus Her2−	4 [20, 22, 25, 29]	50	0.11	F	0.32	0.007
LNM+ Versus LNM−	7 [15–18, 20, 22, 29]	97	<0.00001	R	2.62	0.0001
TNM I/II Versus III/IV	8 [15–18, 22, 26, 28, 29]	96	<0.00001	R	−0.94	0.08
TS 1 Versus 2/3	5 [16, 18, 20, 22, 29]	92	<0.00001	R	−0.58	<0.0001
P53 WT Versus MT	3 [16, 25, 28]	65	0.06	R	−0.79	0.02

*N* number of studies, *P*(*Q*) *P* value of *Q* test for heterogeneity, *P*(*Z*) *P* value of *Z* test for significant test, LNM lymph node metastasis, TS tumor size, WT wild type, MT mutant type.

^a^R: random-effect model; F: fixed-effect model.

## References

[1] Hahn WC, Weinberg RA (2002). Rules for making human tumor cells. *The New England Journal of Medicine*.

[2] Bartel DP (2004). MicroRNAs: genomics, biogenesis, mechanism, and function. *Cell*.

[3] Meister G, Tuschl T (2004). Mechanisms of gene silencing by double-stranded RNA. *Nature*.

[4] Zamore PD, Haley B (2005). Ribo-gnome: the big world of small RNAs. *Science*.

[5] Leva GD, Calin GA, Croce CM (2006). MicroRNAs: fundamental facts and involvement in human diseases. *Birth Defects Research Part C—Embryo Today: Reviews*.

[6] Zhang X, Zhen L, Han X, Shi J, Qiu X, Song W (2012). Expression of three microRNAs in plasma of breast cancer patients. *Chinese Journal of Clinical Oncology*.

[7] Kong W, Yang H, He L (2008). MicroRNA-155 is regulated by the transforming growth factor *β*/Smad pathway and contributes to epithelial cell plasticity by targeting RhoA. *Molecular & Cellular Biology*.

[8] Nam S, Long X, Kwon C, Kim S, Nephew KP (2012). An integrative analysis of cellular contexts, miRNAs and mRNAs reveals network clusters associated with antiestrogen-resistant breast cancer cells. *BMC Genomics*.

[9] Ferlay J, Bray F, Pisani P, Parkin DM (2004). *GLOBOCAN 2002: Cancer Incidence, Mortality and Prevalence Worldwide IARC Cancer Base No 5*.

[10] Cao W, Wang X, Li J (2013). Hereditary breast cancer in the Han Chinese population. *Journal of Epidemiology*.

[11] Atkins D, Best D, Briss PA (2004). Grading quality of evidence and strength of recommendations. *British Medical Journal*.

[12] Higgins JPT, Thompson SG, Deeks JJ, Altman DG (2003). Measuring inconsistency in meta-analyses. *British Medical Journal*.

[13] DerSimonian R, Kacker R (2007). Random-effects model for meta-analysis of clinical trials: an update. *Contemporary Clinical Trials*.

[14] Higgins JPT, Thompson SG (2002). Quantifying heterogeneity in a meta-analysis. *Statistics in Medicine*.

[15] Chen J, Wang B, Tang J (2012). Clinical significance of MicoRNA-155 expression in human breast cancer. *Journal of Surgical Oncology*.

[16] Fu SM, Zhang HW (2011). The expression of miR-155 and miR-21 in breast cancer tissue. *Chinese Journal of Experimental Surgery*.

[17] Hafez MM, Hassan ZK, Zekri ARN (2012). MicroRNAs and metastasis-related gene expression in egyptian breast cancer patients. *Asian Pacific Journal of Cancer Prevention*.

[18] Han XD, Zhang XY, Zhen LL (2013). Circulating miR-155 in serum of patients with breast cancer. *Chinese Journal of Surgical Oncology*.

[19] Heneghan HM, Miller N, Kelly R, Newell J, Kerin MJ (2010). Systemic miRNA-195 differentiates breast cancer from other malignancies and is a potential biomarker for detecting noninvasive and early stage disease. *Oncologist*.

[20] Huang GL, Xue XY, Zeng RC (2013). Expression of microRNAs in plasma of patients with breast cancer and its clinical significance. *Journal of Wenzhou Medical College*.

[21] Iorio MV, Ferracin M, Liu C (2005). MicroRNA gene expression deregulation in human breast cancer. *Cancer Research*.

[22] Lu Z, Ye Y, Jiao D, Qiao J, Cui S, Liu Z (2012). MiR-155 and miR-31 are differentially expressed in breast cancer patients and are correlated with the estrogen receptor and progesterone receptor status. *Oncology Letters*.

[23] Ouyang M, Li Y, Ye S (2014). MicroRNA profiling implies new markers of chemoresistance of triple-negative breast cancer. *Plos ONE*.

[24] Mar-Aguilar F, Mendoza-Ramírez JA, Malagón-Santiago I (2013). Serum circulating microRNA profiling for identification of potential breast cancer biomarkers. *Disease Markers*.

[25] Shao YB, Zhang S, Liu Y (2013). Analysis of serum microRNAs differentially expressed in Breast cancer tissues. *Chinese General Practice*.

[26] Sun Y, Wang M, Lin G (2012). Serum microRNA-155 as a potential biomarker to track disease in breast cancer. *PLoS ONE*.

[27] Wang F, Zheng Z, Guo J, Ding X (2010). Correlation and quantitation of microRNA aberrant expression in tissues and sera from patients with breast tumor. *Gynecologic Oncology*.

[28] Wang XQ, Zhang J (2011). Abnormal expression of miR-155 in serum of breast cancer patients. *Tian Jin Medical College*.

[29] Zheng S, Guo G, Zhang W (2012). Clinical significance of miR-155 expression in breast cancer and effects of miR-155 ASO on cell viability and apoptosis. *Oncology Reports*.

[30] Wiemer EAC (2007). The role of microRNAs in cancer: no small matter. *European Journal of Cancer*.

[31] Silveri L, Tilly G, Vilotte J, Le Provost F (2006). MicroRNA involvement in mammary gland development and breast cancer. *Reproduction Nutrition Development*.

[32] Si ML, Zhu S, Wu H, Lu Z, Wu F, Mo Y-Y (2007). miR-21-mediated tumor growth. *Oncogene*.

[33] Zhu J, Hu X, Guo G (2010). Expression and its clinical significance of miR-155 in human primary breast cancer. *Zhonghua Wai Ke Za Zhi*.

[34] Liu J, Mao Q, Liu Y (2013). Analysis of miR-205 and miR-155 expression in the blood of breast cancer patients. *Chinese Journal of Cancer Research*.

[35] Hui AB, Shi W, Boutros PC (2009). Robust global micro-RNA profiling with formalin-fixed paraffin-embedded breast cancer tissues. *Laboratory Investigation*.

[36] Bian GF, Chen AJ (2012). Correlation study of the expression of miR-21 and miR-155 in the blood and tumor tissue of patients with breast cancer. *Hainan Medical Journal*.

[37] Huang XD, Zhou XR, Li H, Mao L, Liu F (2011). The analysis of expression of miR-155 in Breast cancer tissue and the relationship between the expression and clinical Pathological features. *Chinese Journal of Cancer Prevention and Treatment*.

[38] Allred DC, Harvey JM, Berardo M, Clark GM (1998). Prognostic and predictive factors in breast cancer by immunohistochemical analysis. *Modern Pathology*.

[39] Norberg T, Klaar S, Kärf G, Nordgren H, Holmberg L, Bergh J (2001). Increased p53 mutation frequency during tumor progression—results from a breast cancer cohort. *Cancer Research*.

